# Effects of Selenium Conjugated to Insect Protein on Pharmacokinetics of Florfenicol and Enrofloxacin in Laying Hens

**DOI:** 10.3389/fvets.2021.745565

**Published:** 2021-10-11

**Authors:** Kai Qiu, Uchechukwu Edna Obianwuna, Jing Wang, Hai-jun Zhang, Guang-hai Qi, Shu-geng Wu

**Affiliations:** Risk Assessment Laboratory of Feed Derived Factors to Animal Product Quality Safety of Ministry of Agriculture & Rural Affairs, National Engineering Research Center of Biological Feed, Institute of Feed Research, Chinese Academy of Agricultural Sciences, Beijing, China

**Keywords:** organic selenium, pharmacokinetics, florfenicol, enrofloxacin, laying hens

## Abstract

In the context of increasing awareness on the dietary supplementation of organic selenium in commercial poultry production and ensuring safe egg production, the present study investigated the effects of selenium on the pharmacokinetics of the therapeutic use of florfenicol and enrofloxacin from perspectives of laying performance, selenium deposition in eggs, and drug residue in plasma, organs, and eggs. A 2 × 3 factorial arrangement with two kinds of drugs (florfenicol vs. enrofloxacin, 200 mg/kg) and three levels of dietary organic selenium SCIP (selenium conjugated to insect protein) (0, 2, and 5 mg/kg) was designed together with a blank control group. Healthy Hy-Line Brown laying hens (*n* = 252, 40-week-old and 90.0 ± 1.7% of egg production rate) were randomly allocated into one of seven treatments with six replicates and six hens per replicate. The experiment lasted for 42 days and consisted of three periods (adjusted stage, depositional stage, and eliminating stage) of 14 days each. These stages entail feeding of the laying hens with basal diets, addition of drugs and selenium synchronously into the diets, drug withdrawal from diet, and supply of selenium uninterruptedly in the diet. Egg production and feed intake were recorded on daily and weekly bases, respectively. The selenium content in egg yolk, egg white, and whole eggs and the drug residues in eggs, plasma, liver, kidney, and breast muscle were determined on days 2, 3, 5, 6, 7, 9, 11, and 14 of the depositional and eliminating stages. There was no significant difference (*p* > 0.05) in egg production among the dietary treatments, but feed intake decreased significantly (*p* < 0.05) in the drug treatment group compared to other groups. Dietary organic selenium decreased the residue of drugs in tissues and eggs, while the metabolism and deposition of selenium in laying hens were suppressed due to drug effects. The results of the present study are of significance to enrich the knowledge of the pharmacokinetics of florfenicol and enrofloxacin in laying hens and ensure the quality of poultry products.

## Introduction

In the poultry industry, veterinary drugs such as antibiotics and antiparasitics are extensively used for disease prevention and treatment. Antibiotic growth promoters have been banned on livestock and poultry feed in China in 2020 adhering to the European Union in 2006, and a series of additives were explored to replace antibiotics for the healthy and sustainable development of the poultry industry (1–3). Nonetheless, the occurrences of residues in edible tissues and products caused by therapeutic drugs or contaminated feedstuffs also pose risks to human health, including direct toxic effects, allergic reactions, and increased bacterial resistance to common antibiotics (4). Several medications are approved for poultry production, but relatively few drugs are labeled for laying hens because of the concern of drug residues in chicken eggs (5, 6). Because of the protracted nature of egg development, many weeks may be required before eggs are free of drug residues (7).

Florfenicol and enrofloxacin are wide-spectrum, synthetic antibacterial drugs widely used in the livestock industry, but an adequate drug withdrawal period must be ensured before marketing the animal or its products. In this context, concerns over drug residues in eggs and consumers' health have led several countries including China, UK, and USA to ban the use of florfenicol and enrofloxacin in diets of laying hens (8–10). However, oftentimes, poultry veterinarians have no choice but to use these drugs for treatment from the perspective of animal welfare and therapeutics (6). Drug residues in eggs may also occur once young laying hens are mistakenly or intentionally treated with drugs. The residues of florfenicol and florfenicol amine in eggs of laying hens after oral administration were still detected in eggs within 21 days posttreatment (11). After withdrawal of enrofloxacin oral administration to laying hens, the drug residues decreased but were still detectable in the eggs up to 10 days posttreatment (12). Growth and cardiovascular development are repressed by florfenicol exposure in early chicken embryos (13). In China, the presence of the threshold of drug residues results in unqualified eggs not safe for human consumption. The pharmacokinetics of different veterinary drugs in laying hens and residues in eggs has their own characteristics (14). Therefore, appropriate withdrawal time of therapeutic use of drugs in laying hens is crucial to guaranteeing egg safety and increasing the benefits of the poultry industry.

Nowadays, dietary supplementation of selenium is a common practice in commercial poultry production in order to improve the immunity and overall health of animals (15, 16). Increased intake of dietary selenium for laying hens culminated in a corresponding increase of selenium content in eggs (17). Long-term selenium intake offers a potential therapeutic effect on patients suffering from coronary artery disease (18) and prevents metabolic alterations induced by physical inactivity and sedentary behaviors (19). A new kind of organic selenium, selenium conjugated to insect protein (SCIP) without toxic side effects of the diets (20), was recently exploited to produce safe selenium-enriched eggs. However, the effects of SCIP on pharmacokinetics of common drugs in laying hens have not been investigated. Under the condition of dietary selenium supplementation, the withdrawal time of therapeutic use of florfenicol and enrofloxacin needs to be reconfirmed to ensure egg safety and commercial benefits. This study was conducted to investigate the effects of dietary SCIP on the pharmacokinetics of florfenicol and enrofloxacin from the perspective of laying performance; drug residues in plasma, organs, and eggs; and selenium deposition in eggs.

## Materials and Methods

### Experimental Design, Bird Management, and Sample Collection

A 2 × 3 factorial design with two kinds of drugs (florfenicol vs. enrofloxacin, 200 mg/kg) and three levels of dietary selenium supplementation (0, 2, and 5 mg/kg) was adopted in the present study. The experimental treatments are shown in Table 1. The composition and nutrient levels of the basal diet used in this study are shown in Table 2, which meet or exceed the nutrient requirements of the National Research Council (NRC, 1994) and Chinese Feeding Standard of Chicken (NY/T, 33-2004). The laying hens in the control group (Ctrl) were fed with the basal diet. Selenium was added into experimental diets in the form of SCIP powder. The main steps for the production of SCIP were described as follows. Firstly, selenium-rich bacteria protein was obtained by yeast fermentation using wheat bran and soybean meal as the raw materials supplemented with sodium selenite and low sulfur concentration conditions. Secondly, selenium-rich insect protein was produced by yellow mealworm and super mealworm, taking vegetables and selenium-rich yeast protein as meal. Lastly, SCIP powder was made from SCIP through drying and smashing, and determined to contain 4,480 mg/kg selenium.

**Table 1:** Experimental design.

**Treatments**	**Ctrl**	**FF-0**	**FF-2**	**FF-5**	**EF-0**	**EF-2**	**EF-5**
SCIP, Se, mg/kg	-	-	2	5	-	2	5
FF, 200 mg/kg	-	+	+	+			
EF, 200 mg/kg	-				+	+	+
Calculated Se, mg/kg	0.08	0.08	2.08	5.08	0.08	2.08	5.08
Analyzed Se, mg/kg	0.07	0.07	1.80	5.35	0.07	1.80	5.35

*Ctrl, the control group fed the basal diet; FF, florfenicol; EF, enrofloxacin; SCIP, selenium conjugated to insect protein*.

**Table 2:** The composition and nutrient levels of the basal diet.

**Ingredient,%**	**Contents**	**Nutrients^1^**	**Contents**
Corn	62.96	Crude protein, %	16.50
Soybean meal	26.41	Calcium, %	3.31
Limestone	8.80	Total phosphorus, %	0.54
Dicalcium phosphate	1.00	Available phosphorus, %	0.33
Salt	0.30	AME, MJ/kg	11.29
Premix^2^	0.53	Lysine, %	0.86
		Methionine, %	0.37
Total	100.00	Met+Cys, %	0.65

1 *The values are calculated values. AME, apparent metabolizable energy. ^2^Vitamin and mineral premix provided the following per kg of diets: VA, 12,500 IU; VD_3_, 4,125 IU; VE, 15 IU; VK, 2 mg; VB_1_, 1 mg; VB_2_, 8.5 mg; VB_6_, 8 mg; VB_12_, 5 mg; calcium pantothenate, 50 mg; niacin, 32.5 mg; biotin, 2 mg; folic acid, 5 mg; choline, 500 mg; Mn, 65 mg; I, 1 mg; Fe, 60 mg; Cu, 8 mg; Zn, 66 mg*.

A total of 252 Hy-Line Brown laying hens aged 40 weeks and 90.0 ± 1.7% of egg production rate were randomly allocated into one of seven treatments with six replicates and six hens per replicate. Birds were fed a commercial laying hen diet until the experiment started. Birds had free access to water and diets throughout the experiment and were managed according to the Hy-Line International Online Management Guide (Hy-Line International, 2011). The experiment lasted for 42 days and consisted of three periods (adjusted stage, depositional stage, and eliminating stage) of 14 days, respectively. All the laying hens were fed basal diet at the adjusted stage; the drugs and selenium were synchronously added into diets according to the experimental design at the depositional stage while the drugs were removed from diets to start the eliminating stage with selenium to be supplied uninterruptedly. During the trial, birds were raised in three-tier cages (43 × 43 × 43 cm, three birds per cage) with 16 h of light/day, 20 lx of light intensity, and 14~20°C of room temperature. Egg number, egg weight, abnormal egg, and mortality per replicate were recorded every day. Feed intake per replicate was recorded every week and timely adjusted according to the death of birds. Egg mass was calculated as egg weight × laying rate. The feed conversion ratio (FCR) was calculated as feed intake/egg mass (21). On days 2, 3, 5, 6, 7, 9, 11, and 14 of the depositional and eliminating stages, three eggs per replicate were collected and thoroughly combined as one sample, and the bird with the average body weight of the replicate was selected and fasted for 12 h. After blood sampling from the wing vein, the birds were euthanized and dissected under aseptic conditions. The tissue samples was collected from the liver, kidney, and breast muscle; the plasma was obtained by centrifugation of the blood at 3,000 rpm/min for 15 min at 4°C, and then all samples were stored at−20°C until analysis.

### Chemical Analysis

According to the China National Standard, GB 5009.93-2017, the selenium content in experimental diets, whole egg, albumen, and yolk was analyzed using hydride-atomic fluorescence spectrometry (iCE 3300 AAS, Thermo Fisher Scientific, Rockford, IL, USA) coupled with a standard reference of selenium (GBW8551, National Sharing Platform for Reference Materials, China). The florfenicol residues in eggs and tissues were determined by the high-performance liquid chromatography (HPLC) method in accordance with the recommendations (DB34/T 1376-2011) of the China National Library of Standards. The enzyme-linked immunosorbent assay (ELISA) method was used to analyze the enrofloxacin residue in eggs and tissues in terms of notice no. 1025, 25-2008, published by the Ministry of Agriculture and Rural Affairs of the People's Republic of China.

### Statistical Analysis

Experimental data were analyzed by the one-way ANOVA procedure of SAS 9.2 (SAS Inst. Inc., Cary, NC, USA) for a completely randomized design. The data were also analyzed by 2 × 2 or 2 × 3 factorial using the GLM procedure of SAS for a randomized complete block design. The model included the fixed effect of selenium levels, drugs, and their associated two-way interaction. *p* ≤ 0.05 was set as the threshold for significance.

## Results

### Performance

The performance of laying hens is shown in Table 3. There were no differences between groups for egg production, FCR, and egg weight. The average daily feed intake (ADFI) of laying hens was significantly (*p* ≤ 0.05) affected by experimental treatments. Laying hens fed florfenicol and enrofloxacin (Se-0) showed lower (*p* ≤ 0.05) ADFI than those of the control group, while laying hens fed drugs and selenium were similar with the control group. No interaction effects existed between drugs and selenium levels for egg production, ADFI, FCR, and egg weight of laying hens.

**Table 3:** Effects of dietary organic selenium on performance of laying hens.

**Items^1^**	**Ctrl**	**Florfenicol**			**Enrofloxacin**			**SEM**	***p*** **-value**			
		**Se-0**	**Se-2**	**Se-5**	**Se-0**	**Se-2**	**Se-5**		**A**	**D**	**Se**	**D × S**
**Laying rate, %**
Weeks 1~2	91.27	91.87	93.65	94.64	91.67	93.25	93.06	1.52	0.74	0.60	0.42	0.90
Weeks 3~4	90.87	89.09	93.45	94.65	91.27	92.66	93.46	2.22	0.67	0.97	0.24	0.73
Weeks 5~6	90.48	90.08	91.07	92.26	90.08	92.46	90.68	2.35	0.23	0.85	0.69	0.92
**Egg weight, g**
Weeks 1~2	63.14	62.76	63.54	62.74	63.48	63.12	63.05	0.36	0.67	0.53	0.57	0.37
Weeks 3~4	61.96	61.89	63.25	62.9	62.36	63.22	61.55	0.61	0.29	0.91	0.09	0.30
Weeks 5~6	63.14	62.54	63.4	62.08	62.17	63.45	62.19	0.63	0.33	0.89	0.12	0.92
**Egg mass, kg**
Weeks 1~2	5.76	5.77	5.95	5.94	5.82	5.89	5.87	0.10	0.75	0.75	0.43	0.82
Weeks 3~4	5.63	5.51	5.91	5.95	5.69	5.86	5.75	0.15	0.52	0.94	0.24	0.45
Weeks 5~6	5.71	5.63	5.77	5.73	5.60	5.87	5.64	0.17	0.90	0.83	0.37	0.95
**ADFI, g**
Weeks 1~2	121.26^a^	119.42^b^	121.47^a^	121.16^a^	119.34^b^	120.64^ab^	120.01^ab^	0.61	0.04	0.13	0.01	0.61
Weeks 3~4	117.92	118.46	120.8	117.61	119.82	118.97	119.71	1.37	0.70	0.42	0.35	0.83
Weeks 5~6	119.98	119.89	120.78	116.02	117.18	116.81	118.57	1.76	0.82	0.35	0.67	0.17
**FCR, g/g**
Weeks 1~2	2.11	2.08	2.04	2.04	2.06	2.05	2.05	0.04	0.88	0.96	0.79	0.94
Weeks 3~4	2.11	2.16	2.05	2.06	2.03	2.02	2.08	0.06	0.70	0.59	0.38	0.42
Weeks 5~6	2.11	2.14	2.09	2.03	2.10	2.0.00	2.11	0.06	0.39	0.76	0.55	0.42

*^a, b, c^Means within a row with no common superscripts differ significantly (p <0.05)*.

1 *Data are the mean of six replicates with six birds each; ADFI, average daily feed intake; FCR, feed conversion ratio (feed: egg, g: g); SEM, standard error of mean; A, ANOVA; D, drugs; Se, selenium; Ctrl, the birds fed the basal diet; Se-0, Se-2, and Se-5, the birds fed 0, 2, and 5 mg/kg selenium provided by selenium-rich insect protein powder, respectively. Florfenicol and enrofloxacin were added into experimental diets as 200 mg/kg*.

### Drug Residues in the Body

The drug residues in the plasma, liver, kidney, and breast muscle in the treatment groups are presented as the ratio of their concentration of drugs to those in the control group. As shown in Table 4, the residues of enrofloxacin in plasma were higher (*p* ≤ 0.05) than those of florfenicol during almost all depositional and eliminating stages except for day 7 of the former and day 11 of the latter. At days 5 and 11 of the depositional stage and day 11 of the eliminating stage, residues of both florfenicol and enrofloxacin in plasma were dose-dependently decreased (*p* ≤ 0.05) along with the increase of dietary selenium level. There were no significant interactions between drugs and selenium for the residues of drugs in plasma.

**Table 4:** Effects of dietary organic selenium on residue of drugs in plasma of laying hens.

**Items^1^**	**Florfenicol**			**Enrofloxacin**			**SEM**	***p*** **-value**		
	**Se-0**	**Se-2**	**Se-5**	**Se-0**	**Se-2**	**Se-5**		**D**	**Se**	**D × S**
**Depositional stage**
Day 2	7.47	7.28	7.39	14.02	13.97	13.62	1.09	<0.01	0.89	0.89
Day 3	19.15	17.70	18.08	20.34	20.15	19.77	1.69	0.02	0.56	0.77
Day 5	21.13	17.94	17.21	18.29	17.66	16.57	1.89	0.12	0.02	0.37
Day 7	16.63	17.56	17.08	17.69	17.24	16.25	1.26	0.96	0.46	0.27
Day 9	10.45	9.94	9.76	18.86	18.65	18.67	0.86	<0.01	0.49	0.81
Day 11	14.76	14.32	13.83	21.90	21.00	19.99	1.14	<0.01	0.05	0.67
Day 14	14.88	13.59	14.39	25.53	24.85	23.95	1.37	<0.01	0.21	0.42
**Eliminating stage**
Day 2	16.99	17.09	16.17	19.09	18.83	18.51	1.54	<0.01	0.59	0.92
Day 3	9.55	9.69	9.39	14.49	12.87	14.11	1.07	<0.01	0.34	0.18
Day 5	8.96	8.54	8.25	15.82	14.71	14.79	0.85	<0.01	0.12	0.74
Day 7	8.89	7.87	7.86	10.90	9.42	10.67	1.21	<0.01	0.12	0.56
Day 9	10.92	9.85	10.16	5.72	5.64	5.29	1.10	<0.01	0.44	0.63
Day 11	2.39	1.64	1.49	2.08	1.96	1.37	0.50	0.84	0.01	0.40
Day 14	1.50	1.58	1.35	1.26	1.00	0.92	0.24	<0.01	0.11	0.34

1 *Data are the mean of six replicates calculated by the ratio of drug concentration to the blank control, the birds fed the basal diet; SEM, standard error of mean; D, drugs; Se, selenium; Ctrl, the birds fed the basal diet; Se-0, Se-2, and Se-5, the birds fed 0, 2, and 5 mg/kg selenium provided by selenium-rich insect protein powder, respectively. Florfenicol and enrofloxacin were added into experimental diets as 200 mg/kg*.

The effects of dietary selenium on residues of drugs in the liver of laying hens are presented in Table 5. The residues of florfenicol in the liver was more than (*p* ≤ 0.05) those of enrofloxacin at day 2 of the depositional stage and days 2, 3, 9, 11, and 14 of the eliminating stage, but less (*p* ≤ 0.05) at days 3, 5, 7, 9, and 11 of the depositional stage and day 5 of the eliminating stage. The residues of drugs in the liver at day 3 of the depositional stage and day 7 of the eliminating stage were significantly (*p* ≤ 0.05) affected by dietary selenium levels. There were significant (*p* ≤ 0.05) interactions between drugs and selenium levels at days 3 and 9 of the depositional stage and day 9 of the eliminating stage. At day 3 of the depositional stage, along with the increase of dietary selenium, the residues of florfenicol showed decrease first and then increase (*p* ≤ 0.05), but those of enrofloxacin was not affected. At day 9 of both depositional stage and eliminating stage, the residues of florfenicol were decreased (*p* ≤ 0.05), while those of enrofloxacin were increased (*p* ≤ 0.05), as the dietary selenium levels increased.

**Table 5:** Effects of dietary organic selenium on residue of drugs in liver of laying hens.

**Items^1^**	**Florfenicol**			**Enrofloxacin**			**SEM**	***p*** **-value**		
	**Se-0**	**Se-2**	**Se-5**	**Se-0**	**Se-2**	**Se-5**		**D**	**Se**	**D × S**
**Depositional stage**
Day 2	9.02	8.88	8.68	7.57	7.53	7.43	0.29	<0.01	0.24	0.77
Day 3	14.61	12.24	13.70	17.42	17.23	17.39	0.66	<0.01	<0.01	0.01
Day 5	12.25	12.17	12.41	13.76	13.60	13.47	0.69	<0.01	0.93	0.75
Day 7	14.82	14.28	14.00	15.72	15.58	15.69	0.72	<0.01	0.40	0.50
Day 9	14.91	14.39	14.09	14.90	15.23	18.37	1.46	<0.01	0.07	0.01
Day 11	16.05	15.06	15.10	19.65	17.30	20.66	1.83	<0.01	0.11	0.19
Day 14	16.72	16.02	14.57	17.00	15.09	16.16	1.63	0.61	0.11	0.26
**Eliminating stage**
Day 2	16.70	15.77	15.19	14.43	14.34	13.70	1.31	<0.01	0.21	0.76
Day 3	14.92	13.83	13.52	10.80	10.77	11.09	0.86	<0.01	0.40	0.21
Day 5	6.90	6.78	6.49	10.81	10.14	10.39	0.84	<0.01	0.53	0.76
Day 7	5.80	5.82	5.43	6.61	6.56	5.17	0.57	0.08	0.01	0.14
Day 9	6.55	6.48	5.41	2.63	2.83	2.87	0.49	<0.01	0.08	0.02
Day 11	4.77	4.88	4.33	1.40	1.24	1.11	0.43	<0.01	0.14	0.55
Day 14	3.31	2.72	3.10	1.32	1.11	1.27	0.47	<0.01	0.27	0.73

1 *Data are the mean of six replicates calculated by the ratio of drug concentration to the blank control, the birds fed the basal diet; SEM, standard error of mean; D, drugs; Se, selenium; Ctrl, the birds fed the basal diet; Se-0, Se-2, and Se-5, the birds fed 0, 2, and 5 mg/kg selenium provided by selenium-rich insect protein powder, respectively. Florfenicol and enrofloxacin were added into experimental diets as 200 mg/kg*.

Residues of drugs in the kidney of laying hens are shown in Table 6. At days 2, 5, 11, and 14 of the depositional stage and days 3 and 5 of the eliminating stage, the residues of florfenicol were less (*p* ≤ 0.05) than those of enrofloxacin, but were more (*p* ≤ 0.05) at day 14 of the eliminating stage. The dietary selenium levels did not affect the residues of drugs, and no interactions were observed between drugs and selenium levels. As shown in Table 7, the residues of drugs in breast muscle of laying hens were significantly different between florfenicol and enrofloxacin. At days 2, 3, 5, 7, 9, and 14 of the depositional stage and days 2, 5, 9, and 14 of the eliminating stage, the residues of florfenicol were more (*p* ≤ 0.05) than those of enrofloxacin, but less (*p* ≤ 0.05) at day 11 of the depositional stage and days 3 and 11 of the eliminating stage. Along with the increase of dietary selenium, the residues of drugs were significantly decreased (*p* ≤ 0.05) at day 3 of the depositional stage and days 2 and 11 of the eliminating stage. There were no significant interactions between drugs and dietary selenium levels during the depositional and eliminating stages.

**Table 6:** Effects of dietary organic selenium on residue of drugs in kidney of laying hens.

**Items^1^**	**Florfenicol**			**Enrofloxacin**			**SEM**	***p*** **-value**		
	**Se-0**	**Se-2**	**Se-5**	**Se-0**	**Se-2**	**Se-5**		**D**	**Se**	**D × S**
**Depositional stage**
Day 2	8.74	8.66	8.47	9.64	9.61	9.56	0.24	<0.01	0.30	0.71
Day 3	10.64	9.10	9.74	9.38	9.26	9.22	0.81	0.16	0.22	0.33
Day 5	8.05	7.76	7.74	11.28	11.09	10.62	0.57	<0.01	0.24	0.70
Day 7	11.62	11.39	10.82	11.36	11.07	10.84	0.83	0.55	0.23	0.90
Day 9	15.16	14.33	14.45	14.43	13.85	13.35	1.12	0.10	0.25	0.85
Day 11	9.19	9.06	8.78	15.49	15.06	14.99	0.85	<0.01	0.53	0.93
Day 14	13.32	13.21	13.25	17.34	17.02	15.80	0.85	<0.01	0.17	0.20
**Eliminating stage**
Day 2	14.14	14.21	14.56	14.26	13.52	13.75	0.84	0.16	0.65	0.43
Day 3	10.55	10.22	10.49	12.97	12.80	12.79	0.75	<0.01	0.79	0.93
Day 5	10.49	10.38	10.55	9.74	9.16	9.18	0.78	<0.01	0.62	0.67
Day 7	7.10	6.37	6.14	6.51	5.98	5.89	0.84	0.22	0.14	0.92
Day 9	5.16	5.03	3.86	4.60	4.05	4.08	1.01	0.29	0.19	0.48
Day 11	2.89	2.33	2.11	2.35	2.26	2.27	0.53	0.48	0.22	0.38
Day 14	2.04	1.79	1.81	1.64	1.61	1.57	0.28	0.02	0.48	0.74

1 *Data are the mean of six replicates calculated by the ratio of drug concentration to the blank control, the birds fed the basal diet; SEM, standard error of mean; D, drugs; Se, selenium; Ctrl, the birds fed the basal diet; Se-0, Se-2, and Se-5, the birds fed 0, 2, and 5 mg/kg selenium provided by selenium-rich insect protein powder, respectively. Florfenicol and enrofloxacin were added into experimental diets as 200 mg/kg*.

**Table 7:** Effects of dietary organic selenium on residue of drugs in breast muscle of laying hens.

**Items^1^**	**Florfenicol**			**Enrofloxacin**			**SEM**	***p*** **-value**		
	**Se-0**	**Se-2**	**Se-5**	**Se-0**	**Se-2**	**Se-5**		**D**	**Se**	**D × S**
**Depositional stage**
Day 2	5.17	5.13	5.13	2.04	2.00	1.98	0.09	<0.01	0.46	0.95
Day 3	5.38	4.50	4.53	3.68	3.23	2.64	0.65	<0.01	0.01	0.59
Day 5	7.27	7.05	7.23	4.41	4.26	4.05	0.49	<0.01	0.67	0.71
Day 7	9.54	9.66	9.57	8.55	8.41	8.32	0.55	<0.01	0.92	0.87
Day 9	16.00	15.19	14.88	14.35	14.00	13.27	1.07	<0.01	0.10	0.87
Day 11	14.09	13.87	13.50	15.78	15.26	15.28	0.97	<0.01	0.52	0.91
Day 14	16.07	15.50	15.21	14.86	14.36	14.33	0.79	<0.01	0.19	0.90
**Eliminating stage**
Day 2	15.85	15.30	15.13	12.20	11.75	11.14	0.65	<0.01	0.04	0.78
Day 3	9.13	8.96	9.04	11.01	10.74	9.96	0.64	<0.01	0.24	0.31
Day 5	10.52	10.32	10.19	8.82	7.88	8.39	0.80	<0.01	0.35	0.60
Day 7	6.96	7.07	6.78	6.50	6.50	6.04	0.74	0.07	0.58	0.94
Day 9	6.81	6.64	6.22	3.70	3.86	3.80	0.68	<0.01	0.68	0.57
Day 11	1.97	2.03	1.81	2.41	2.48	1.82	0.33	0.02	0.01	0.27
Day 14	2.32	1.75	2.10	1.58	1.51	1.45	0.40	<0.01	0.33	0.48

1 *Data are the mean of 6 replicates calculated by the ratio of drug concentration to the blank control, the birds fed the basal diet; SEM, standard error of mean; D, drugs; Se, selenium; Ctrl, the birds fed the basal diet; Se-0, Se-2, and Se-5, the birds fed 0, 2, and 5 mg/kg selenium provided by selenium-rich insect protein powder, respectively. Florfenicol and enrofloxacin were added into experimental diets as 200 mg/kg*.

### Drug Residues in Eggs

The drug residues of eggs in the treatment groups are presented as the ratio of their concentration of drugs in eggs to those in the control group (Table 8). The residue rate of enrofloxacin in eggs was significantly higher (*p* ≤ 0.05) than that of florfenicol at the whole depositional stage and days 2, 3, 5, and 11 of the eliminating stage. For laying hens fed florfenicol, diets supplemented with 2 mg/kg selenium significantly (*p* ≤ 0.05) decreased the drug residue rate in eggs at day 2 of the depositional stage relative to the control group, while those with 5 mg/kg selenium showed significant (*p* ≤ 0.05) opposite effects. The residue rate of florfenicol in eggs for laying hens fed 2 mg/kg selenium was significantly (*p* ≤ 0.05) decreased as compared with the control group at days 3 and 14 of the eliminating stage, that of 5 mg/kg selenium was significantly (*p* ≤ 0.05) decreased at day 2, and that of 2 mg/kg selenium was less (*p* ≤ 0.05) than that of 5 mg/kg at day 7. At days 2 and 11 of the eliminating stage, the residue rate of enrofloxacin in eggs showed significantly (*p* ≤ 0.05) gradient reduction along with increased level of dietary selenium. During the whole depositional stage, no interaction effects existed between drugs and selenium levels for drug residue of eggs. At day 2 of the eliminating stage, significant (*p* ≤ 0.05) interaction effects on drug residue were observed between drugs and selenium and disappeared during the rest of the eliminating stage.

**Table 8:** Effects of dietary organic selenium on residue of drugs in eggs of laying hens.

**Items^1^**	**Florfenicol**			**Enrofloxacin**			**SEM**	***p*** **-value**		
	**Se-0**	**Se-2**	**Se-5**	**Se-0**	**Se-2**	**Se-5**		**D**	**Se**	**D × S**
**Depositional stage**
Day 2	4.06^b^	3.61^c^	4.45^a^	10.37	10.35	10.04	0.23	<0.01	0.60	0.14
Day 3	6.15	6.41	5.89	16.00^x^	15.69^xy^	14.86^y^	0.55	<0.01	0.59	0.84
Day 5	7.41	7.29	6.87	16.46	14.19	15.05	0.48	<0.01	0.23	0.34
Day 7	7.65	7.02	7.02	17.34	15.77	16.78	0.81	<0.01	0.63	0.89
Day 9	7.48	6.84	7.50	14.96	14.34	15.19	0.77	<0.01	0.79	1.00
Day 11	7.68	7.75	7.62	18.90	19.31	17.81	0.62	<0.01	0.64	0.72
Day 14	7.25	7.02	7.12	20.70	20.44	19.29	0.55	<0.01	0.53	0.59
**Eliminating stage**
Day 2	5.91^a^	5.57^ab^	5.27^b^	13.41^x^	11.44^y^	10.16^z^	0.27	<0.01	<0.01	<0.01
Day 3	2.64^a^	2.36^b^	2.57^ab^	7.89	7.65	7.59	0.26	<0.01	0.77	0.92
Day 5	1.59	1.47	1.51	2.34	2.39	2.34	0.05	<0.01	0.75	0.36
Day 7	1.17^ab^	1.11^b^	1.19^a^	1.34	1.22	1.09	0.06	0.37	0.34	0.22
Day 9	1.07	1.00	1.01	1.10	1.07	0.94	0.04	0.76	0.15	0.43
Day 11	1.12	1.03	1.04	1.41^x^	1.24^y^	1.09^z^	0.05	<0.01	<0.01	0.12
Day 14	1.13^a^	1.03^b^	1.07^ab^	1.09	1.10	1.09	0.04	0.74	0.71	0.59

1 *Data are the mean of six replicates calculated by the ratio of drug concentration to the blank control, the birds fed the basal diet; SEM, standard error of mean; D, drugs; Se, selenium; Ctrl, the birds fed the basal diet; Se-0, Se-2, and Se-5, the birds fed 0, 2, and 5 mg/kg selenium provided by selenium-rich insect protein powder, respectively. Florfenicol and enrofloxacin were added into experimental diets as 200 mg/kg. a–c and x–z. Means in a row with no common superscripts within one drug are significantly different (P <0.05)*.

### Selenium Enriched in Eggs

The concentration of selenium in the yolk of eggs is shown in Table 9. Laying hens fed diets supplemented with selenium had a higher (*p* ≤ 0.05) concentration of selenium in the yolk of eggs than those in the control group from day 5 of the depositional stage to the end of the eliminating stage. Increasing dietary selenium from 2 to 5 mg/kg significantly (*p* ≤ 0.05) increased the content of selenium in yolk of laying hens fed enrofloxacin at days 7 and 9 of the depositional stage, while no effects were observed at other time points of the depositional and eliminating stages. Dietary selenium levels showed no effects on the selenium enriched in yolk of laying hens fed florfenicol during the depositional and eliminating stages, except for day 5 of the latter where the dietary selenium content exhibited a positive relationship (*p* ≤ 0.05) with the concentration of selenium in yolk. For laying hens fed 5 mg/kg selenium, enrofloxacin increased (*p* ≤ 0.05) the enrichment of selenium in yolk relative to florfenicol. During the two stages, significant (*p* ≤ 0.05) interaction effects were observed only at day 9 of the depositional stage.

**Table 9:** Effects of dietary organic selenium and drugs on the concentration of selenium in egg yolk of laying hens.

**Items^1^, μg/100g**	**Ctrl**	**Florfenicol**		**Enrofloxacin**		**SEM**	***p*** **-value**			
		**Se-2**	**Se-5**	**Se-2**	**Se-5**		**A**	**D**	**Se**	**D × S**
**Depositional stage**
Day 2	36.83	37.00	37.00	37.33	36.67	1.01	0.99	1.00	0.74	0.74
Day 3	36.83	36.50	37.33	36.83	38.50	0.88	0.74	0.51	0.27	0.71
Day 5	38.5^b^	58.00^a^	60.67^a^	59.33^a^	63.50^a^	1.27	<0.01	0.31	0.10	0.71
Day 7	36.17^c^	83.00^a^	84.33^a^	77.00^b^	82.83^a^	0.80	<0.01	0.01	0.01	0.09
Day 9	35.33^c^	104.17^ab^	101.33^ab^	100.50^b^	105.83^a^	1.01	<0.01	0.80	0.44	0.02
Day 11	35.83^b^	104.50^a^	104.00^a^	105.00^a^	107.5^a^	1.30	<0.01	0.12	0.31	0.20
Day 14	37.17^c^	107.00^ab^	108.83^a^	105.33^b^	106.67^ab^	0.70	<0.01	0.06	0.11	0.80
**Eliminating stage**
Day 2	37.67^b^	106.17^a^	106.83^a^	105.33^a^	107.67^a^	0.97	<0.01	1.00	0.17	0.44
Day 3	35.00^b^	106.83^a^	107.83^a^	106.17^a^	109.00^a^	1.67	<0.01	0.92	0.43	0.70
Day 5	35.17^c^	110.83^a^	104.17^b^	108.33^ab^	109.50^ab^	1.67	<0.01	0.55	0.25	0.11
Day 7	35.50^b^	106.67^a^	107.33^a^	106.33^a^	105.67^a^	1.33	<0.01	0.60	1.00	0.73
Day 9	32.50^c^	107.50^ab^	105.17^b^	107.17^ab^	109.33^a^	1.03	<0.01	0.17	0.95	0.11
Day 11	34.00^b^	108.00^a^	104.17^a^	108.33^a^	105.83^a^	1.61	<0.01	0.66	0.17	0.77
Day 14	34.33^b^	107.00^a^	104.83^a^	107.00^a^	108.00^a^	1.36	<0.01	0.42	0.76	0.42

*^a, b, c^Means within a row with no common superscripts differ significantly (p <0.05)*.

1 *Data are the mean of six replicates. SEM, standard error of mean; A, ANOVA; D, drugs; Se, selenium; Ctrl, the birds fed the basal diet; Se-2 and Se-5, the birds fed 2 and 5 mg/kg selenium provided by selenium-rich insect protein powder, respectively. Florfenicol and enrofloxacin were added into experimental diets as 200 mg/kg*.

The content of selenium enriched in albumen of eggs is presented in Table 10. Dietary selenium significantly (*p* ≤ 0.05) increased the concentration of selenium in albumen relative to the control group from day 5 of the depositional stage to the end of the eliminating stage. At day 2 of the eliminating stage, the dietary selenium level showed positive effects (*p* ≤ 0.05) on the enrichment of selenium in albumen. For laying hens fed florfenicol, at day 7 of the depositional stage, the diet containing 5 mg/kg selenium significantly (*p* ≤ 0.05) increased the content of selenium in albumen as compared with that of 2 mg/kg selenium. Increasing dietary selenium from 2 to 5 mg/kg obviously (*p* ≤ 0.05) increased the enriched selenium in albumen of eggs for laying hens fed enrofloxacin. Drugs and dietary selenium levels exhibited significant (*p* ≤ 0.05) interaction effects on the content of selenium in albumen on day 2 of the depositional stage.

**Table 10:** Effects of dietary organic selenium and drugs on the concentration of selenium in egg albumen of laying hens.

**Items^1^, μg/100g**	**Ctrl**	**Florfenicol**		**Enrofloxacin**		**SEM**	***p*** **-value**			
		**Se-2**	**Se-5**	**Se-2**	**Se-5**		**A**	**D**	**S**	**D × S**
**Depositional stage**
Day 2	5.53	5.68	5.52	5.53	5.83	0.09	0.15	0.35	0.46	0.02
Day 3	5.52	5.73	5.55	5.60	5.72	0.09	0.43	0.87	0.75	0.16
Day 5	5.45^b^	7.60^a^	7.45^a^	7.48^a^	7.53^a^	0.17	<0.01	0.94	0.81	0.63
Day 7	5.63^c^	43.83^b^	47.33^a^	46.83^ab^	48.33^a^	0.82	<0.01	0.10	0.04	0.40
Day 9	5.32^b^	73.50^a^	74.67^a^	74.00^a^	76.17^a^	1.16	<0.01	0.54	0.31	0.76
Day 11	5.60^b^	79.83^a^	78.50^a^	77.17^a^	80.17^a^	0.97	<0.01	0.71	0.54	0.12
Day 14	5.80^b^	77.00^a^	79.17^a^	77.17^a^	78.00^a^	0.75	<0.01	0.65	0.19	0.55
**Eliminating stage**
Day 2	5.35^c^	75.67^ab^	80.33^a^	73.83^b^	77.67^ab^	1.29	<0.01	0.26	0.04	0.83
Day 3	5.68^b^	76.67^a^	78.00^a^	75.33^a^	78.67^a^	0.98	<0.01	0.81	0.11	0.48
Day 5	5.71^b^	76.67^a^	78.67^a^	75.50^a^	77.33^a^	1.04	<0.01	0.41	0.21	0.96
Day 7	5.73^c^	76.83^ab^	78.33^ab^	76.17^b^	79.33^a^	0.76	<0.01	0.88	0.04	0.45
Day 9	5.82^b^	76.33^a^	79.67^a^	75.83^a^	78.00^a^	1.03	<0.01	0.50	0.10	0.72
Day 11	5.80^b^	76.83^a^	77.17^a^	77.00^a^	78.50^a^	1.05	<0.01	0.66	0.59	0.73
Day 14	5.81^b^	77.17^a^	78.17^a^	76.83^a^	78.83^a^	0.94	<0.01	0.90	0.27	0.71

*^a, b, c^Means within a row with no common superscripts differ significantly (p <0.05)*.

1 *Data are the mean of six replicates. SEM, standard error of mean; A, ANOVA; D, drugs; Se, selenium; Ctrl, the birds fed the basal diet; Se-2 and Se-5, the birds fed 2 and 5 mg/kg selenium provided by selenium-rich insect protein powder, respectively. Florfenicol and enrofloxacin were added into experimental diets as 200 mg/kg*.

The yolk and albumen of the egg were mixed together evenly, and then the selenium content of the mixture was determined (Table 11). It is the same with those in yolk and albumen that the concentration of selenium in the whole egg was significantly increased by dietary selenium supplementation relative to the control group from day 5 of the depositional stage to the end of the eliminating stage. At day 7 of the depositional stage and days 2 and 7 of the eliminating stage, increasing dietary selenium levels from 2 to 5 mg/kg significantly elevated the content of selenium in eggs. Laying hens fed two kinds of drugs showed similar selenium contents in eggs. There were no interaction effects between drugs and dietary selenium levels on the enrichment of selenium in eggs at the depositional and eliminating stages.

**Table 11:** Effects of dietary organic selenium and drugs on the concentration of selenium in whole egg of laying hens.

**Items^1^, μg/100g**	**Ctrl**	**Florfenicol**		**Enrofloxacin**		**SEM**	***p*** **-value**			
		**Se-2**	**Se-5**	**Se-2**	**Se-5**		**A**	**D**	**Se**	**D × S**
**Depositional stage**
Day 2	14.23	14.39	14.26	14.37	14.4	0.28	0.99	0.84	0.88	0.80
Day 3	14.21	14.28	14.38	14.28	14.83	0.29	0.67	0.50	0.32	0.49
Day 5	14.63^b^	21.61^a^	22.24^a^	21.89^a^	23.09^a^	0.44	<0.01	0.31	0.11	0.61
Day 7	14.12^c^	54.72^b^	57.62^a^	55.22^b^	57.92^a^	0.67	<0.01	0.65	<0.01	0.91
Day 9	13.47^b^	82.02^a^	82.08^a^	81.37^a^	84.41^a^	0.97	<0.01	0.51	0.23	0.25
Day 11	14.00^b^	86.69^a^	85.59^a^	84.90^a^	87.76^a^	0.85	<0.01	0.87	0.45	0.10
Day 14	14.51^c^	85.34^ab^	87.41^a^	84.99^b^	85.97^ab^	0.59	<0.01	0.26	0.06	0.48
**Eliminating stage**
Day 2	14.33^c^	84.14^ab^	87.70^a^	82.59^b^	85.54^ab^	0.99	<0.01	0.20	0.03	0.83
Day 3	13.83^b^	85.05^a^	86.29^a^	83.90^a^	86.63^a^	0.80	<0.01	0.73	0.10	0.52
Day 5	13.90^b^	86.17^a^	85.75^a^	84.63^a^	86.27^a^	0.78	<0.01	0.65	0.58	0.36
Day 7	14.00^c^	85.12^ab^	86.39^ab^	84.55^b^	86.65^a^	0.49	<0.01	0.82	0.02	0.54
Day 9	13.23^b^	84.99^a^	86.75^a^	84.54^a^	86.24^a^	0.81	<0.01	0.68	0.14	0.98
Day 11	13.63^b^	85.49^a^	84.67^a^	85.71^a^	85.63^a^	0.86	<0.01	0.66	0.73	0.78
Day 14	13.74^b^	85.46^a^	85.58^a^	85.22^a^	86.47^a^	0.74	<0.01	0.75	0.50	0.58

*^a, b, c^Means within a row with no common superscripts differ significantly (p <0.05)*.

1 *Data are the mean of six replicates. SEM, standard error of mean; A, ANOVA; D, drugs; Se, selenium; Ctrl, the birds fed the basal diet; Se-2 and Se-5, the birds fed 2 and 5 mg/kg selenium provided by selenium-rich insect protein powder, respectively. Florfenicol and enrofloxacin were added into experimental diets as 200 mg/kg*.

## Discussion

The organic selenium SCIP as a new kind of selenium source conjugated to insect protein has been demonstrated to be beneficial for the health of laying hens and improve the safety of selenium enriched eggs (20). In the poultry industry, laying hens are inevitably exposed to some drugs owing to routine or intentional therapeutic schemes (6). Appropriate withdrawal time of drugs in laying hens is crucial to guaranteeing that drug residues are completely absent in eggs, since conventional methods of egg cooking cannot eliminate all veterinary drug residues (22). Selenium could enhance the antioxidant defense system of the body and thereby maintain the normal physiology and optimal health of animals (23). Therefore, evaluation of the effects of SCIP on the pharmacokinetics of common poultry drugs, florfenicol and enrofloxacin, in laying hens is of great significance in the poultry industry and consumers.

Recently, various studies have investigated and confirmed the positive effects of different selenium sources on the laying performance and antioxidant capacity of laying birds including sodium selenite, nanoselenium, selenomethionine, and selenium yeast (24–32). In the current study, the selenium obtained through two steps of biotransformation including microbial fermentation and insect synthesis is of higher bioactivity and biosafety than traditional selenium sources (20). During the trial period, no significant improvement was observed in the performance of the laying hens due to drug (florfenicol and enrofloxacin) effect; this could be accrued to the healthy status of the birds and an indication that drug therapy was not needful. Drugs and selenium have no interactive effects on the performance of layers during the adjusted stage, depositional stage, and eliminating stage of drugs. However, the feed intake of laying hens decreased significantly due to drug effects; such could probably be due to the non-palatability of the diets caused by the drugs (33). Se supplementation increased the feed intake of laying hens in the current study, which is consistent with the previous reports that there was a higher feed intake with selenium levels (24).

Eggs from laying hens have been reported to contain drug residues from different classes of drugs used in the poultry for therapeutic purposes, and such residues can persist for a long time in the posttreatment period (14). In the present study, the residues of florfenicol in the plasma, liver, kidney, breast muscle, and eggs of laying hens were significantly different from that of enrofloxacin during the depositional and eliminating stages. This affirms that there is a conspicuous variation in the pharmacokinetics of these drugs in laying hens. For instance, the elimination time for levamisole residue completely after oral administration in laying hens is longest in liver samples, followed by thigh muscle, breast muscle, kidney, and eggs (34). The residues of tapentadol in eggs after multiple oral dose administration in laying hens were higher in yolk than those in albumen (35). After florfenicol withdrawal, 57% of the drugs were eliminated from laying hens through the egg yolk (9). In the current study, elimination of residues of florfenicol and enrofloxacin was fastest in the egg followed by plasma while the retention time of florfenicol and enrofloxacin residue was longest in the liver and kidney, respectively. After treatment of laying hens with enrofloxacin, the residues decreased but were still detectable in egg white and yolk at day 8 and day 10, respectively, in the posttreatment period (12). Therefore, the residues of florfenicol and enrofloxacin in yolk and albumen of eggs need to be studied in detail separately. The enrofloxacin peaks gradually increased until the fifth day of successive administration (36). In the present study, the concentration of enrofloxacin in plasma and liver hits a plateau on the second day of continuous oral administration, while those in kidney and eggs rise continuously. Selenium is an essential nutrient and crucial to humans' metabolic activity, where selenoproteins act as antioxidant tools for thyroid regulation and anti-inflammatory action (37). In the current study, dietary organic selenium significantly decreased the residue of drugs in the plasma, liver, breast muscle, and eggs of laying hens, which is probably due to the regulated action of selenoproteins. Therefore, we deduce that selenium probably decreases the residue of drugs through enhancing the metabolism of laying hens. No interaction was found between drugs and selenium on the residue of drugs in laying hens, which indicated that selenium enhances the metabolism of laying hens without being affected by the kind of drugs.

Increased intake of dietary selenium culminated in a corresponding increase of selenium content in eggs and meat (17). It is consistent with the results of our study that the concentration of selenium in yolk, albumen, or the whole egg increased with supplementation of organic selenium in the diet of laying hens. Increasing the level of selenium supplementation in laying hens' diet during the period at which florfenicol and enrofloxacin were in use did not culminate in increasing the deposition of selenium in eggs in the current study. It is inconsistent with our previous results that the content of selenium deposited in eggs increased along with the increased dietary SCIP supplementation (20), an indication that the homeostatic balance between selenium metabolism and other elements required for the biological functions of the laying hens has been distorted due to drug effect (38). Besides, no interaction effects between dietary selenium level and types of drugs on the concentration of selenium in eggs were observed, an implication that drugs used in the current study are irrespective of the type of suppressed selenium metabolism and its deposition in the eggs of laying hens.

## Conclusion

Dietary organic selenium decreases the residue of drugs in tissues and eggs probably through enhancing the metabolism of laying hens. The treatment of drugs showed inhibition effects on the metabolism and deposition of selenium in laying hens. In the context of the wide application of selenium in poultry feed, these findings contribute to the knowledge of the pharmacokinetics of florfenicol and enrofloxacin in laying hens and ensuring the safety of poultry products.

## Data Availability Statement

The raw data supporting the conclusions of this article will be made available by the authors, without undue reservation.

## Ethics Statement

The animal study was reviewed and approved by Animal Care and Use Committee of the Feed Research Institute of the Chinese Academy of Agricultural Sciences.

## Author Contributions

Conceptualization, methodology, and project administration were performed by S-gW and G-hQ. Animal experiment, chemical analysis, and data collection were conducted by KQ and H-jZ. Original draft was written by KQ. Writing-review was finished by H-jZ, JW, and UO. All authors have agreed to the final manuscript.

## Funding

This study was supported by the earmarked fund for Modern Agro-industry Technology Research System (CARS-40-K12), Beijing Innovation Consortium of Agriculture Research System (BAIC04-2021), Shandong Key Science and Technology Innovation Program (2019JZZY010704), National Natural Science Foundation of China (32072774), and Agricultural Science and Technology Innovation Program (ASTIP) of the Chinese Academy of Agricultural Sciences.

## Conflict of Interest

The authors declare that the research was conducted in the absence of any commercial or financial relationships that could be construed as a potential conflict of interest.

## Publisher's Note

All claims expressed in this article are solely those of the authors and do not necessarily represent those of their affiliated organizations, or those of the publisher, the editors and the reviewers. Any product that may be evaluated in this article, or claim that may be made by its manufacturer, is not guaranteed or endorsed by the publisher.
